# Impact of Severity of COVID-19 in TB Disease Patients: Experience from an Italian Infectious Disease Referral Hospital

**DOI:** 10.3390/idr17010011

**Published:** 2025-02-05

**Authors:** Virginia Di Bari, Carlotta Cerva, Raffaella Libertone, Serena Maria Carli, Maria Musso, Delia Goletti, Alessandra Aiello, Antonio Mazzarelli, Angela Cannas, Giulia Matusali, Fabrizio Palmieri, Gina Gualano, on behalf of the TB-INMI Working Group

**Affiliations:** 1 Respiratory Infectious Diseases Unit, National Institute for Infectious Diseases “Lazzaro Spallanzani”, IRCCS, 00149 Rome, Italy; virginia.dibari@inmi.it (V.D.B.); raffaella.libertone@inmi.it (R.L.); serena.carli@inmi.it (S.M.C.); maria.musso@inmi.it (M.M.); fabrizio.palmieri@inmi.it (F.P.); gina.gualano@inmi.it (G.G.); 2 Translational Research Unit, National Institute for Infectious Diseases (INMI) “Lazzaro Spallanzani”, IRCCS, 00149 Rome, Italy; delia.goletti@inmi.it (D.G.); alessandra.aiello@inmi.it (A.A.); 3 Microbiology and Bio-Repository, National Institute for Infectious Diseases (INMI) “Lazzaro Spallanzani”, IRCCS, 00149 Rome, Italy; antonio.mazzarelli@inmi.it (A.M.); angela.cannas@inmi.it (A.C.); 4 Laboratory of Virology and Biosafety Laboratories, National Institute for Infectious Diseases “Lazzaro Spallanzani”, IRCCS, 00149 Rome, Italy; giulia.matusali@inmi.it

**Keywords:** tuberculosis, COVID-19, TB-COVID-19 coinfection, SARS-CoV-2, TB disease, TB severity, COVID-19 severity

## Abstract

Background/Objectives: Tuberculosis (TB) remains a major global health issue, further complicated by the COVID-19 pandemic. This study assesses the clinical outcomes of TB-COVID-19-coinfected patients compared to those with TB disease alone at an Italian infectious disease hospital during the pandemic’s first two years. Methods: Retrospective data analysis was conducted on TB patients hospitalized from March 2020 to June 2022. Data included demographics, comorbidities, clinical characteristics, and outcomes. Coinfection was defined as concurrent TB disease and SARS-CoV-2 infection. Statistical methods included Fisher’s exact test and Mann–Whitney statistics. Results: Of 267 TB patients, 25 (9.4%) had concurrent COVID-19 infection. The TB-COVID-19 group showed higher rates of diabetes and cough. Acute respiratory failure was more prevalent in coinfected patients (odds ratio, 5.99), and coinfection was associated with worse outcomes compared to TB alone (odds ratio, 0.15). Despite similar socio-demographic factors, the coexistence of TB and COVID-19 led to exacerbated respiratory failure and increased mortality. Conclusions: Coinfection with TB and COVID-19 significantly increases the risk of acute respiratory failure and poor outcomes. Clinicians should be aware of this risk, especially in patients with pulmonary involvement. Although specific protocols are unavailable, prompt diagnosis and management may enhance outcomes. Additional research is necessary to understand the long-term effects of TB-COVID-19 coinfection, particularly as COVID-19 becomes endemic.

## 1. Introduction

Tuberculosis disease (TB) remains a significant global health concern, with an estimated 8.2 million new cases and 1.25 million deaths worldwide in 2023 [1].

In Europe, the WHO reported 33.52 thousand new cases and 20 thousand deaths in 2021. In the same year, Italy recorded 2480 new TB disease cases, a notification rate of 4.0% per 100,000 population [2].

The emergence of the Severe Acute Respiratory Syndrome Coronavirus 2 (SARS-CoV-2) disease (COVID-19) pandemic has necessitated a robust public health response owing to its rapid global transmission, severe clinical manifestations, and high mortality rate [3,4,5].

Owing to the COVID-19 disease, an excess of 14.83 million deaths was globally estimated during the 2020–2021 period [6].

The COVID-19 pandemic has significantly impacted TB services. Several studies have reported a decrease in TB disease diagnoses and case management, and an increasing trend toward more severe clinical presentations during the pandemic [7,8,9,10,11,12,13,14].

The WHO reported an 18% global decrease in TB disease case notifications from 2019 to 2020 (from 7.1 to 5.8 million cases) [15].

Studies have noted an exacerbation of both diseases when co-existing, attributed to common social, epidemiological, and clinical determinants [16,17,18].

Both TB disease and COVID-19 primarily affect the lungs [19], presenting similar clinical symptoms (cough, fever, fatigue, dyspnea), making differential diagnosis challenging. Both diseases also share complications, including respiratory failure and thromboembolism [20].

In 2021, the CDC published a brief review examining the interactions between TB and COVID-19 before the initiation of the COVID-19 vaccination program [21]. The review concluded that TB-COVID-19 coinfection was associated with increased COVID-19 mortality. In 2022, the TB/COVID-19 Global Study Group network published data from the first cohort of TB-COVID-19-coinfected patients. The multi-country study reported a 12% mortality rate and an increased risk of TB disease.

This study aims to characterize the clinical features and outcomes of TB/COVID-19-coinfected individuals in the first two years of the pandemic and compare these findings with a cohort of “only TB disease” patients treated at an Italian TB-COVID-19 referral hospital.

## 2. Materials and Methods

### 2.1. Study Design and Participants

We conducted a retrospective data collection and analysis of all patients consecutively hospitalized for TB disease at INMI from March 2020 to June 2022. Data were obtained from the local TB database, approved by the L. Spallanzani Institute Ethics Committee (Decision No. 12/2015), with patients providing written informed consent for the use of anonymized clinical data.

**Figure 1:**
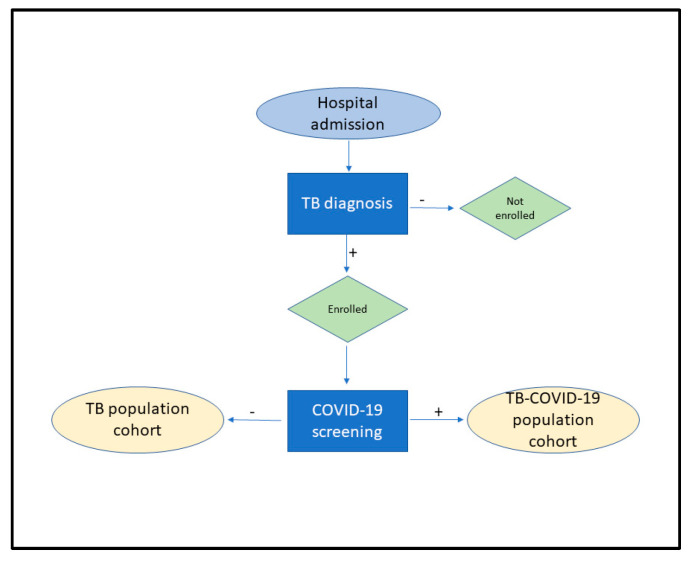
Patient inclusion flowchart.

A patient inclusion flowchart is shown in Figure 1.

We collected socio-demographic data (sex, age, nationality), comorbidities, and clinical and microbiological characteristics (symptoms, drug resistance pattern, concurrent extrapulmonary TB disease, and outcome). Respiratory failure was defined as arterial oxygen pressure (PaO2) less than 60 mmHg (8.0 kPa) when breathing room air.

Radiological severity of TB disease at admission was also recorded, with extensive (or advanced) TB disease defined as the presence of bilateral cavitary disease or extensive parenchymal damage on chest radiography [22].

Treatment outcome was evaluated until September 2024, with definitions based on the World Health Organization (WHO) tuberculosis treatment guidelines fourth edition [23].

At hospital admission, all patients underwent molecular nasopharyngeal swabbing testing RT-PCR for SARS-CoV-2. Coinfection was defined as a concurrent diagnosis of TB disease alongside SARS-CoV-2 infection, while superinfection refers to SARS-CoV-2 diagnosed in a patient already with TB disease before admission.

### 2.2. Statistical Analysis

Categorical variables were summarized using counts and percentages, and medians and interquartile ranges were used for continuous variables. Risk factors were assessed using Fisher’s exact test for categorical variables and Mann–Whitney two-sample statistics for quantitative variables. All tests were two-sided, and *p*-values < 0.05 were considered significant. Data were analyzed using Stata software, release 15.0 (Stata Corp, College Station, TX, USA).

An ordered logistic regression model was used to quantify the impact of COVID-19 infection on both the severity of illness and the outcomes among individuals diagnosed with tuberculosis (TB). This approach enables us to assess the relationship between co-infection and progression of disease severity across ordered categorical outcomes.

The estimated coefficient quantifies the effect of a one-unit change in the COVID-19 variable on the likelihood of an unfavorable outcome. This allows us to calculate the increased probability of experiencing an unfavorable outcome for COVID-positive patients compared to non-COVID patients

## 3. Results

Between March 2020 and June 2022, 267 cases of TB disease were consecutively admitted at the INMI hospital. The median age was 44 (IQR: 54–32), and 156 (58.4%) patients had one or more comorbidities. Two hundred and thirty-six (88.4%) cases were TB microbiologically confirmed, while diagnosis in 31 cases was based on clinical–radiological (17 cases; 6.4%) or histological criteria (14; 5.2%). For 34 patients, it was a relapse of TB disease after a previously cured disease. The median length of stay was 20 (range 0–121) days.

Twenty-five out of 267 (9.4%) patients had SARS-CoV-2 infection at admission. Among this population, 10 (40.0%) patients had a prior TB disease diagnosis, while 15 (60.0%) patients had concurrent new diagnoses of TB disease and COVID-19. Six TB-COVID-19 cases were TB disease relapses, eleven (44%) had radiological signs of COVID-19 pneumonia, and ten (40%) had cavitary lesions at the TC scan at admission. Only two patients were vaccinated against SARS-CoV-2 infection, both with boosters. Only one case received early COVID-19 therapy with an antiviral drug (Remdesivir) while the other four patients received other drugs under clinical trials (e.g., recombinant IL-1 receptor antagonist).

Regarding the follow-up, 188/267 (70.4%) patients were followed-up until cure or later, 9 (3.4%) patients died (4 patients during the stay in hospital and 5 patients during the follow-up period), and 66 (24.7%) were lost at follow-up or transferred to another center.

Table 1 presents the characteristics of the total population and TB or TB-COVID-19 population.

There were no significant differences in socio-demographic factors, infection site, or microbiological findings between TB and TB-COVID-19 groups. However, diabetes was more common in the TB-COVID-19 group (*p* = 0.017), and coughing was a more frequent symptom (*p* < 0.001).

In terms of clinical features, in the entire population, 70 out of 267 (26.2%) patients had acute respiratory failure treated with oxygen supplement or non-invasive ventilation, and one patient needed mechanical ventilation and was admitted to ICU. In the TB-COVID-19 group, the percentage of patients with acute respiratory failure was 64% (16/25). Multiple logistic regression revealed that acute respiratory failure was significantly different between TB and TB-COVID-19 populations (odds ratio, 5.99; CI, 0.02 to 0.92). Furthermore, TB-COVID-19 coinfection was significantly associated with a worse outcome than TB single disease (odds ratio, 0.15; CI, 0.02 to 0.92).

## 4. Discussion

In our study, we analyzed a cohort of TB-COVID-19-coinfected patients compared with TB-mono-infected patients in terms of demographical and clinical features, during the SARS-CoV-2 pandemic.

In our study, no significant differences were observed between TB and TB-COVID-19 populations in terms of socio-demographic factors, infection site, or microbiological findings. Cough was significantly more prevalent in the TB-COVID-19-coinfected group vs. TB single disease, and comorbidities were more prevalent in the TB-COVID-19 population (*p* = 0.017).

A more severe picture of respiratory failure was found in the coinfected population (odds ratio, 5.99; CI, 0.02 to 0.92) and TB-COVID-19 coinfection was significantly associated with a worse outcome (odds ratio, 0.15; CI, 0.02 to 0.92).

The COVID-19 pandemic has had a multifaceted impact on TB control efforts globally. Limited access to healthcare has hindered TB detection, delayed diagnosis, and impaired treatment. The coexistence of COVID-19 and TB presents complex challenges, requiring comprehensive strategies for effective management [24].

Even though COVID-19 also requires droplet and contact precautions compared with TB, which is an airborne-only pathology, both diseases share similarities in their transmission mechanisms and primarily affect the respiratory system, making pulmonary involvement a critical aspect of their pathophysiology [19]. TB disease can cause extensive destruction of lung tissue and form caverns, leading to chronic cough and other respiratory symptoms, as well as impaired respiratory function. Similarly, SARS-CoV-2 damages lung cells, triggering inflammation and compromising respiratory function, causing severe pneumonia and acute respiratory distress syndrome (ARDS) [25,26,27,28,29,30,31].

A more severe picture of respiratory failure and a worse outcome found in our TB-COVID-19 population are findings similar to other recent studies and reviews.

Following published scientific evidence in the literature, TB disease was listed by the CDC as the underlying medical condition associated with a higher risk for severe COVID-19 after the CDC systematic review process. GB Migliori et al. with the Global Tuberculosis Network and TB/COVID-19 Global Study Group published data on long-term outcomes from the largest cohort of coinfected patients. Factors independently associated with overall mortality were age, supplemental oxygen needed, and invasive ventilation [32].

A systematic review identified coinfection as a risk factor for COVID-19 severity and TB disease progression [33]. Sarkar S et al. published a meta-analysis in 2021 focusing on the impact of COVID-19 on patients with concurrent coinfections. The mortality rate among TB disease patients coinfected with COVID-19 was significantly higher compared to the control group (RR = 2.10, 95% CI: 1.75 to 2.51, I2 = 0%) [34].

In patients with TB-COVID-19 coinfection living in countries with the highest burdens of TB, malnutrition and low body mass index (BMI) have been recognized as major risk factors for early mortality [35]. In our population, no difference was found in terms of nutritional status.

The immune response in individuals coinfected with COVID-19 and TB is intricate [36]. As for SARS-CoV-2, the control of TB infection requires a timely innate response as well as an effective adaptive response [19,36,37]. COVID-19 and TB share similar immune response imbalances, potentially worsening disease progression when coinfected [38,39] but the clinical and immunopathological interaction between TB and COVID-19 and the drivers of dual disease mortality are not yet fully understood [36,40,41,42].

Our results confirm that TB patients are more susceptible to severe COVID-19 disease.

The development of broad and coordinated antigen-specific adaptive immune responses to SARS-CoV-2 is essential for controlling COVID-19. The immune response to *M. tuberculosis* is primarily mediated by Th1 responses. The interaction between *M. tuberculosis*-specific and SARS-CoV-2-specific immune responses remains incompletely understood. In a study published in 2021, the authors investigated the impact of concurrent infections with SARS-CoV-2 and *M. tuberculosis* on the immune responses specific to each pathogen, utilizing a whole-blood assay platform. The study concluded that patients co-infected with TB and COVID-19 have a reduced capacity to develop an immune response to SARS-CoV-2. This finding suggests that TB impairs the ability to mount a SARS-CoV-2-specific immune response in co-infected individuals [36].

Current evidence indicates that patients with TB infection may experience enhanced immune modulation against COVID-19. In contrast, those with TB disease might exhibit a diminished specific immune response to SARS-CoV-2 and reduced lymphocyte functionality, potentially leading to ineffective infection control [43,44].

Our study population is young (median age 44 y), and most of the patients were foreigners and socially vulnerable. Data from the USA CDC have shown that people from racial and ethnic minority groups are more likely to be infected with SARS-CoV-2 and, once infected, they are more likely to be hospitalized, be admitted to the ICU, and die from COVID-19 at younger ages [45].

Vaccination for SARS-CoV-2 could mitigate COVID-19 in TB-coinfected patients. In our population, the TB vaccination rate was very low because in Italy vaccinations for SarsCov2 started in December 2020 for high-risk groups (health workers, elderly). After that period the vaccination strategy was implemented to voluntary acceptance and free of charge for all people. As stated, our study population was younger and socially vulnerable, with possible low adherence to the vaccination campaign.

Most researchers consider pulmonary TB disease a risk factor for severe COVID-19 infection, with several studies suggesting that hyperinflammation or impaired ability to mount a SARS-CoV-2-specific immune response in coinfected subjects could contribute to unfavorable outcomes [36,46,47].

A recent U.S. study involving 333 coinfected TB-COVID-19 patients reported a twofold increase in mortality, corroborating the findings of multiple case-control studies that validate the altered disease pathology in coinfections [48]. Moreover, several researchers indicate an increased risk of TB infection transitioning into TB disease in the context of COVID-19 due to the depletion of CD4 + T cells [38,49,50].

Coinfection may impair a specific response to SARS-CoV-2 and *M. tuberculosis* [36,37].

Up to date, in most of the studies, the patients included in the report were at different TB stages, spanning from TB infection, TB disease, cured TB, and TB disease and TB under treatment. In our study, all patients have a diagnosis of TB disease (TB and past or cured TB were not included). It is important to restrict our analysis to evaluate the impact of coinfection on clinical course and prognosis, rather than the effect of a respiratory virus infection like SARS-CoV-2 on lungs previously affected by another infectious disease or with a TB infection that has not yet progressed to disease.

Our study population is homogeneous concerning COVID-19 immunization and preventive therapy: only two patients were vaccinated for SARS-CoV2, and only one received early treatment with Remdesivir. Sixty percent had extensive TB disease, highlighting the COVID-19 pandemic’s impact on TB care, including diagnostic delays, severe clinical presentations, and outcomes. We note that a recent systematic review did not find a significant association between SARS-CoV-2 coinfection and unfavorable TB treatment outcomes [51].

Long-term follow-up care is essential for individuals recovering from coinfection because both diseases can have long-lasting effects on respiratory function. In our population, more than two-thirds of individuals have successfully transitioned from the acute phase of coinfection to completing TB treatment.

Research on the residual functional impairment following coinfection with COVID-19 and TB is currently limited. Coinfection could lead to exacerbated lung damage, resulting in persistent respiratory symptoms, such as chronic cough, shortness of breath, and reduced lung capacity [52]. In a small study, it was reported that coinfected patients have twice the risk of spirometry abnormalities than the general population [53].

Given the limited data, further research is essential to comprehensively understand the long-term functional outcomes of COVID-19 and TB coinfection. This knowledge is crucial for developing effective rehabilitation strategies and improving patient care.

Italy was the first European country to be affected by the COVID-19 pandemic. Our center, as the Italian reference center for tuberculosis and, with the outbreak of the pandemic, also for COVID-19, received, especially in the first two years, patients from other centers with SARS-CoV-2 infection and patients already followed by our outpatient clinic for TB disease or for suspected TB. This peculiarity meant that the relevant population was screened early for both diseases with a good diagnostic yield. We can perhaps consider this a bias in that in our reality access to care was certainly easier than in high endemic countries where, as is well known and published in the literature, the COVID-19 pandemic caused global disruptions to health services, with well-documented negative impacts on TB-infected patients and TB-related services.

Limitations of this work should be accounted for. The retrospective nature of the study did not allow us to consider additional factors potentially influencing the outcome of patients, such as other chronic disorders or other risk factors for severe COVID-19. In addition, in our study, COVID-19 was diagnosed either because patients had clinical signs of SARS-CoV-2 infection or through an active screening program, and COVID-19 diagnosis was based only on the results of real-time RT-PCR for SARS-CoV-2 from nasopharyngeal swabs. Although the data were carefully collected and the analysis results robust, our findings may have limited generalizability as the patient sample was from a single center.

Finally, the sample size difference between TB and TB-COVID-19 cases could raise concerns about variability and be a significant limitation of this study.

## 5. Conclusions

Based on our experience, coinfection with TB disease and COVID-19 is significantly associated with a higher prevalence of acute respiratory failure and poorer patient outcomes. Clinicians should be aware of this potentially life-threatening situation, particularly in patients who exhibit pulmonary involvement indicative of both diseases and need to adopt unique clinical strategies, including enhanced monitoring and management of immune responses, tailored treatment plans that address the interactions between TB and COVID-19 therapies, increased vigilance for potential complications and comprehensive patient education and support to ensure adherence to treatment regimens.

Further research is needed to evaluate TB-COVID-19 coinfection, especially now that COVID-19 has become endemic.

## Figures and Tables

**Table 1:** Population characteristics.

		Total Population (=267)	TB Population (=242)	TB-COVID-19 Population (=25)	*p*-Value
		N (%)	N (%)	N (%)	
Gender	Male Female	179 (67.0) 88 (33.0)	158 (65.3) 84 (34.7)	21 (84.0) 4 (16.0)	0.058
Nationality	South American African European Asian	18 (6.7) 40 (15.0) 155 (58.1) 54 (20.2)	15 (6.2) 36 (56.2) 145 (18.6) 46 (19.0)	3 (12.0) 4 (16.0) 10 (40.0) 8 (32.0)	0.404
Smoking	Yes	95 (35.6)	86 (35.5)	9 (36)	0.992
Comorbidity	Chronic alcoholism Diabetes Liver disease Chronic respiratory diseases Immunodeficiency Malnutrition	45 (16.9) 27 (10.1) 53 (19.9) 78 (29.2) 25 (9.4) 43 (16.1)	38 (15.7) 21 (8.7) 47 (19.4) 73 (30.2) 21 (8.7) 39 (16.1)	7 (28.0) 6 (24.0) 6 (24.0) 5 (20.0) 4 (16.0) 4 (16.0)	0.123 0.017 0.827 0.277 2.231 0.958
Infection site	Lung Lung and extrapulmonary Extrapulmonary TB	168 (62.9) 73 (27.3) 26 (9.7)	153 (63.2) 65 (26.8) 24 (9.9)	15 (60.0) 8 (32.0) 2 (8.0)	0.750 0.583 0.758
Symptoms	Fever Cough Fatigue/malaise Weight loss Hemoptysis	91 (34.1) 182 (68.2) 81 (30.3) 78 (29.2) 32 (12)	78 (32.2) 172 (71.1) 75 (31.0) 67 (27.7) 31 (12.8)	13 (52.0) 10 (40.0) 6 (24.0) 11 (44.0) 1 (4.0)	0.059 0.001 0.424 0.105 0.184
Pts with microbiological diagnosis	Sputum smear positive Sputum molecular positive test Sputum culture positive	143 (53.6) 91 (34.1) 2 (0.7)	130 (53.7) 81 (33.5) 2 (0.8)	13 (52.0) 10 (50.0) 0 (0.0)	0.853 0.475 0.816
	Drug-resistance	24 (9.0)	21 (8.7)	3 (12.0)	0.677
Extensive TB disease	Yes	163 (61.0)	155 (64.0)	8 (32.0)	0.204
Acute respiratory failure	Yes	70 (26.2)	54 (22.3)	16 (64.0)	0.001
Outcome	Recovery Death	181 (67.8) 4 (1.5)	164 (67.8) 2 (0.8)	17 (68.0) 2 (8.0)	0.0049

## Data Availability

All relevant data are within the manuscript. Raw data are accessible, if requested, from the National Institute for Infectious Diseases “L. Spallanzani” Library to the E-mail address: biblioteca@inmi.it.
